# Supplementary material for: Site-specific photolabile roadblocks for the study of transcription elongation in biologically complex systems
Source: Commun Biol. 2022 May 12;5:457. doi: 10.1038/s42003-022-03382-0 (PMC9098449; doi:10.1038/s42003-022-03382-0)
Supplement: Supplementary file 2 — Supplementary Information [file 42003_2022_3382_MOESM2_ESM.pdf]

Site-specific photolabile roadblocks for the study of transcription elongation in biologically complex systems

Jean-François Nadon[1#], Vitaly Epshtein[2,3], Etienne Cameron[4#], Mikhail R. Samatov[5], Andrey S. Vasenko[5], Evgeny Nudler[2,3] and Daniel A. Lafontaine[1]*

[1]Department of Biology, Faculty of Science, RNA Group, Université de Sherbrooke, Sherbrooke, Quebec, Canada, J1K 2R1.

[2]Department of Biochemistry and Molecular Pharmacology, New York University School of Medicine, New York, NY 10016, USA.

[3]Howard Hughes Medical Institute, New York University School of Medicine, New York, NY 10016, USA.

[4]Department of Chemical Engineering, Polytechnique Montreal, Quebec, Canada, H3T 1J4.

[5]HSE University, 101000 Moscow, Russia.

[#]Present address: Pancosma Canada Inc, Drummondville, Quebec, Canada, J2C 7V5.

*Corresponding author. E-mail: daniel.lafontaine@usherbrooke.ca

Keywords: transcriptional pausing; transcriptional roadblock; elongation complex; NPOM.

Running title: NPOM as a removable transcriptional roadblock

SUPPLEMENTARY INFORMATION

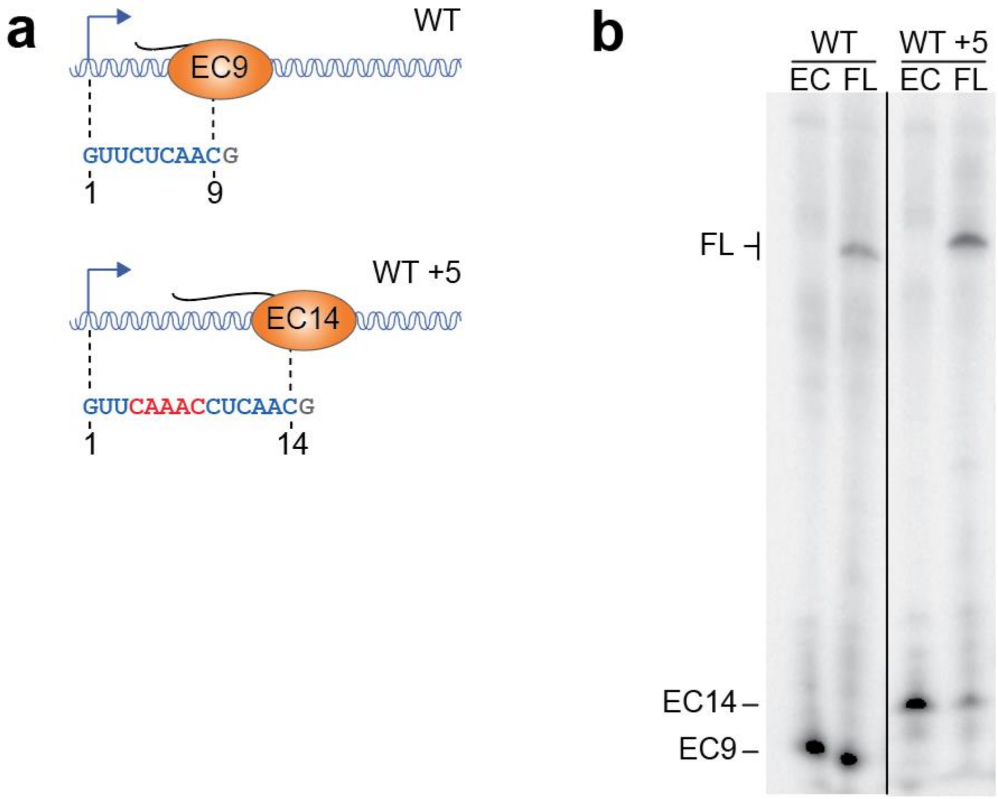

Supplementary Figure 1. *In vitro* transcription reactions performed using the *Escherichia coli tbpA* riboswitch.

(**a**) Schematic representing the formation of EC9 (top) and EC14 (bottom). While EC9 is produced using the wild-type riboswitch, EC14 is obtained by incorporating five nucleotides within the sequence (indicated in red). For both EC9 and EC14, transcription reactions are performed using GUU, ATP, UTP and CTP. The omission of GTP does not allow RNAP to incorporate guanosine (indicated in light gray).

(**b**) Transcription reactions using the natural sequence (WT) and the 5- nucleotide extension (WT +5). EC9 and EC14 were obtained by adding GUU, ATP, [α-^{32}P] UTP and CTP. The full length (FL) was obtained by purifying EC9 or EC14 using G50 columns and by adding 1 mM of NTP. The production of EC9 appears to produce more abortive transcripts that do not restart upon NTP addition, compared to EC14 (compare EC to FL products for both WT and WT +5 constructs). The experiments were performed at least two times and variations were less than 10%.

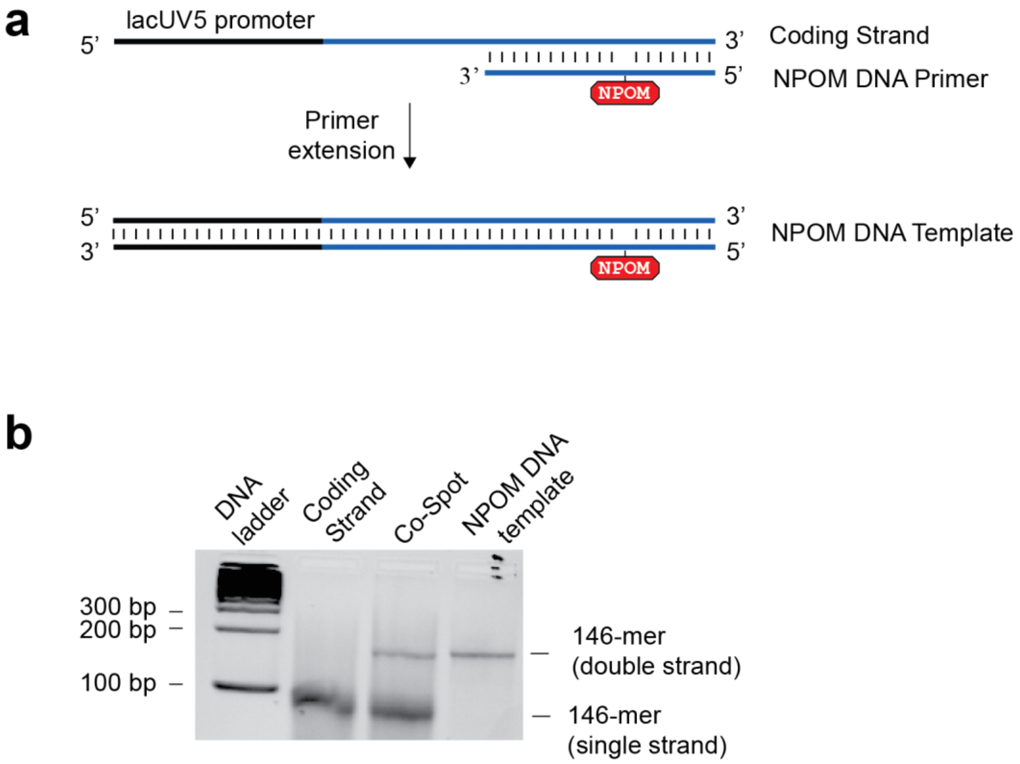

Supplementary Figure 2. Preparation of the NPOM DNA template.

(**a**) Schematic of the primer extension approach used to prepare the NPOM *tbpA* DNA template. A reverse oligonucleotide containing an NPOM-caged dT at the 8th position was used as a primer to allow extension by the Taq DNA polymerase. The forward full-length single strand ultramer (146 nucleotides) was used as a template.

(**b**) Agarose gel (4%) stained with ethidium bromide showing the results of the primer extension compared to the single strand template. The starting single strand ultramer (coding strand) and products of the primer extension reaction were loaded on the gel. A "co-spot" lane was used to show that both the single strand ultramer and primer extension products were well resolved during the migration. After two temperature cycles, disappearance of the single stranded template and formation of double stranded NPOM DNA were observed. The experiments were performed at least two times and variations were less than 10%.

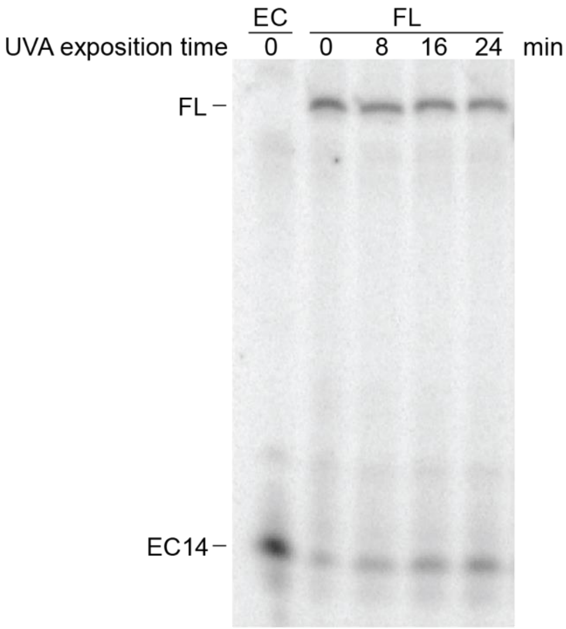

Supplementary Figure 3. Effect of UVA illumination on stalled ECs at position 14.

In vitro transcribed EC14 were illuminated with UVA for various times. After illumination, NTP were added to the reaction mixture to resume transcription elongation. Even after 24 minutes of UVA exposition, transcription elongation proceeds to yield full-length products, indicating that the UVA light does not prevent ECs to resume transcription. The experiments were performed at least two times and variations were less than 10%.

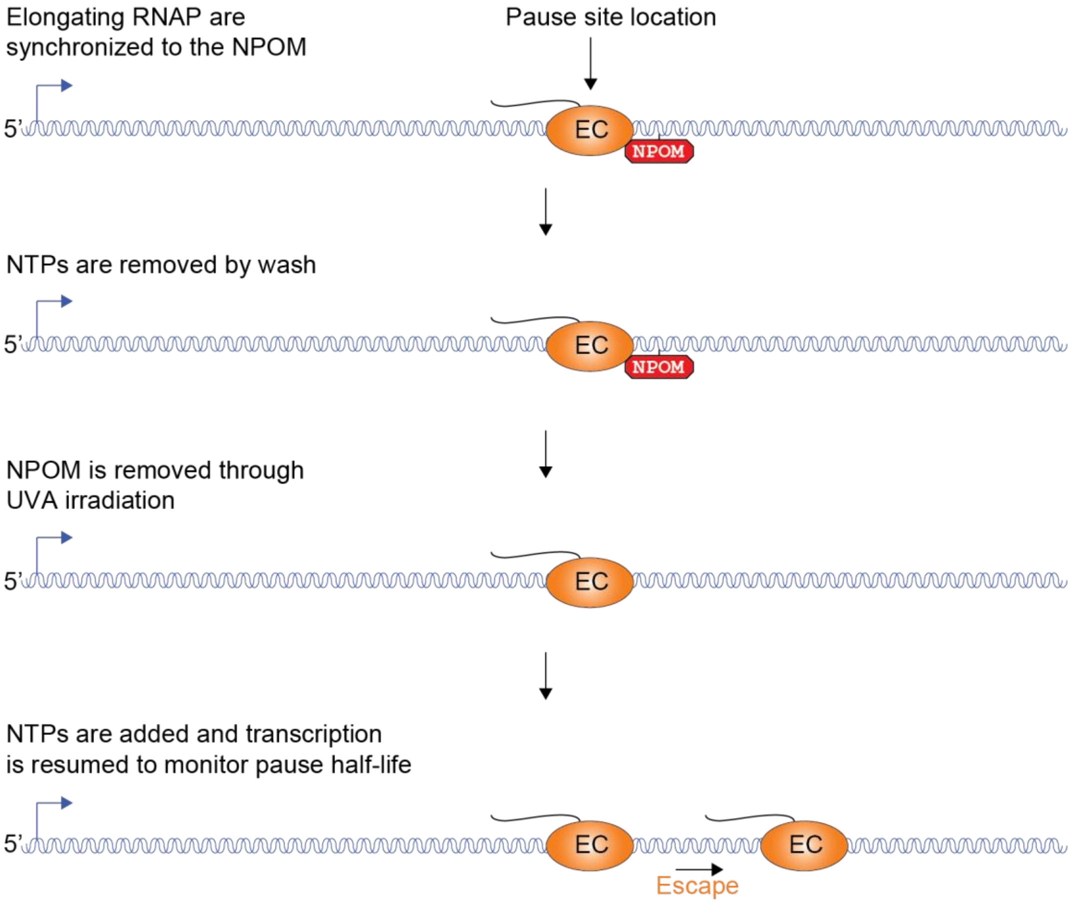

Supplementary Figure 4. Kinetics of transcription elongation using NPOM templates.

In the first step of the procedure, elongation complexes are allowed to reach the NPOM roadblock, thus resulting in the synchronization of RNAPs at the NPOM site. In the second step, unincorporated NTPs are washed using G50 columns. The third step involves UVA irradiation of ECs to remove the NPOM roadblock. In the last step, transcription elongation can be resumed by adding NTPs and the pause half-life can thus be monitored.

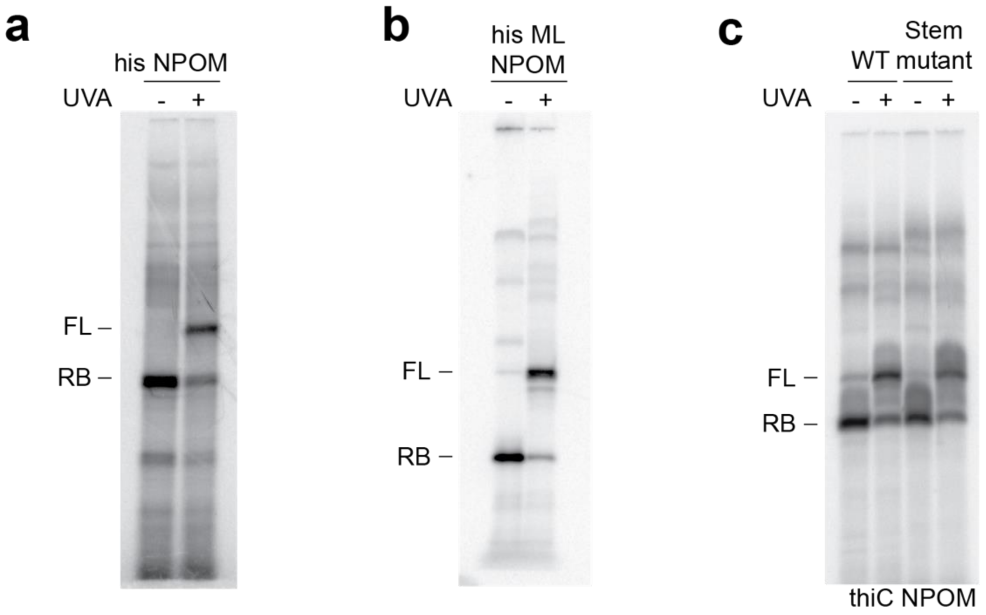

Supplementary Figure 5. Single-round transcription assays of NPOM-containing DNA templates.

(**a**) Transcription reactions performed using the *his* NPOM construct. In the absence of UVA light (-), elongating complexes are stalled at the roadblock (RB), thus generating a 101-nt transcript. Upon exposing to UVA light (+), transcription elongation was resumed to yield the full-length (FL) products. The experiments were performed at least two times and variations were less than 10%.

(**b**) Transcription reactions performed using the minimal length *his* NPOM construct. The roadblock (RB) species corresponds to a 40-nt transcript. Exposition to UVA light (+) allows transcription reactions to resume, thus yielding the full-length product (FL).

(**c**) Transcription reactions performed using the *thiC* NPOM construct. The roadblock (RB) product corresponds to a transcript of ~70-nt and exposition to UVA light (+) permits the production of the full-length transcript (FL). Experiments were performed using the wild-type (WT) and a A138 stem mutant (G119C, G120C, C121G, G122C). NPOM removal was performed in the presence of NTPs for panels (a-c).

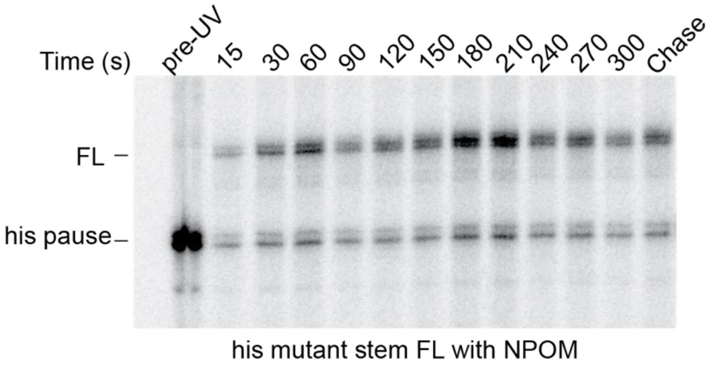

Supplementary Figure 6. Transcription kinetics experiments monitoring the *his* pause half-life using the NPOM stem mutant template.

Transcription reactions were performed using 150 µM UTP, CTP and GTP, and 10 µM ATP. Aliquots of the reaction were taken at the indicated time. A chase reaction was also done. A large fraction of full-length products is observed at 15 s, suggesting that the stem mutant disrupts transcriptional pausing. NPOM removal was performed in the absence of NTPs. The experiments were performed at least two times and variations were less than 10%.

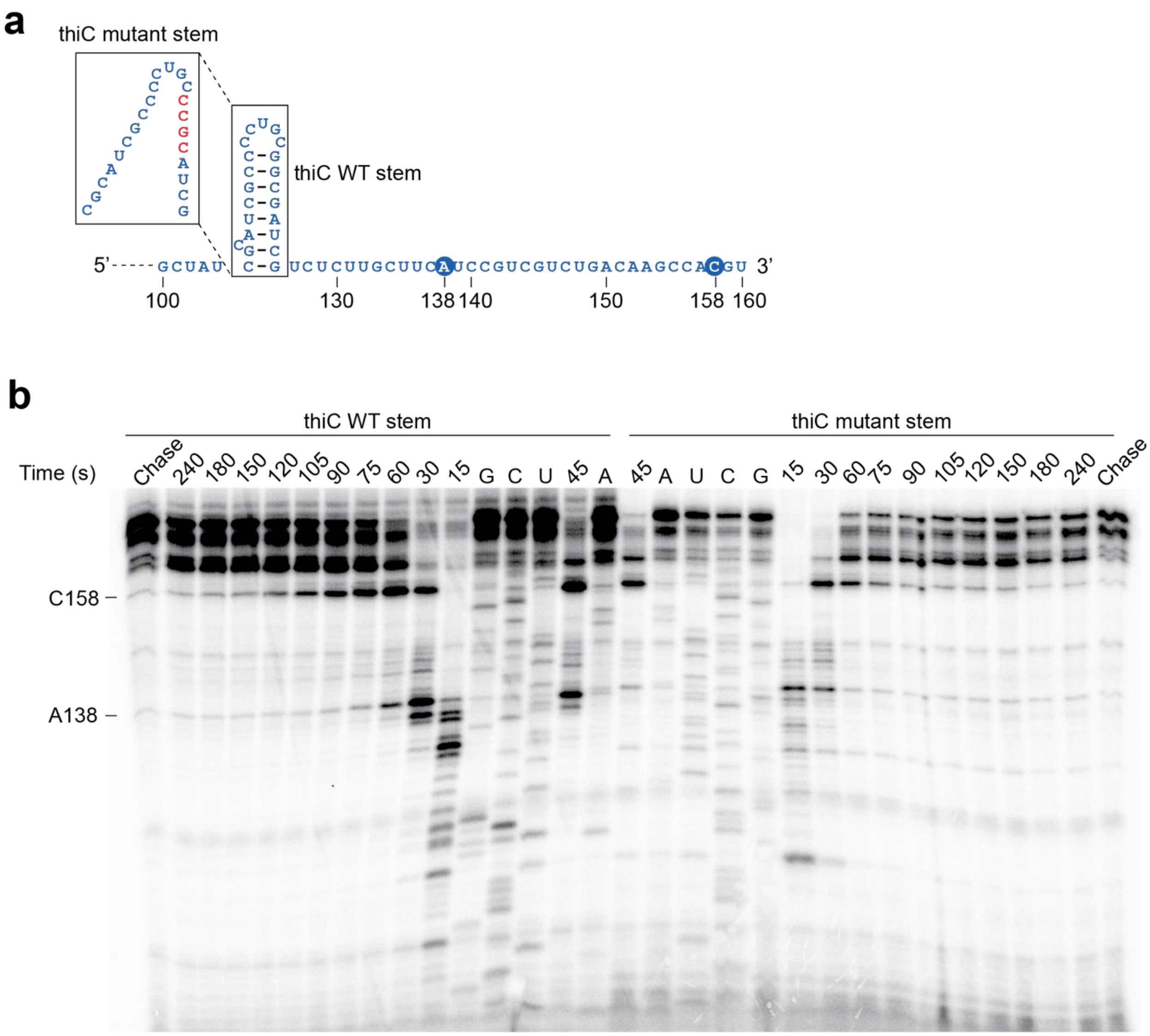

Supplementary Figure 7. Mapping of pause sites using *thiC* 87-175 templates.

(**a**) RNA sequence of *thiC* 87-175 in the context of the wild-type and the stem mutant. The mutations introduced to destabilize the A138 stem are shown in red. The pause sites A138 and C158 are shown in blue circles.

(**b**) Mapping of pause sites using 3'-OMe RNA sequencing reactions. For each transcription, the 45 s sample, containing a population of ECs paused at the A138 as well as the C158 pause sites, was loaded next to the ladders. Aliquots of reactions were taken at the indicated time and chase reactions were also performed. Experiments were performed for the wild-type and the A138 mutant stem. The experiments were performed at least two times and variations were less than 10%.

Supplementary Table 1. Half-life values determined in this study.

Row	Template	Pause	NusA	Half-lives
1	*pLacUV5-his-132-WT (FL)*	U102	-	37 ± 5 s
2	*pLacUV5-his-132-WT (FL)*	U102	+	127 ± 5 s
3	*pLacUV5-his-132-G103A (FL)*	U102	-	44 ± 2 s
4	*pLacUV5-his-132-NPOM (FL)*	U102	-	12 ± 2 s
5	*pLacUV5-his-132-NPOM (FL)*	U102	+	44 ± 5 s
6	*pLacUV5-his-132-NPOM-StemMut (FL)*	U102	-	N/A
7	*pLacUV5-his-62-132 (ML)*	U102	-	13 ± 1 s
8	*pLacUV5-his-62-132 (ML)*	U102	+	20 ± 2 s
9	*pLacUV5-his-62-132 NPOM (ML)*	U102	-	12 ± 2 s
10	*pLacUV5-his-62-132 NPOM (ML)*	U102	+	18 ± 2 s
11	*pLacUV5-thiC-87-175-G159A*	A138	-	16 ± 2 s
12	*pLacUV5-thiC-87-175-G159A*	C158	-	27 ± 2 s
13	*pLacUV5-thiC-87-175-G159A-StemMut*	A138	-	8 ± 1 s
14	*pLacUV5-thiC-87-175-G159A-StemMut*	C158	-	16 ± 4 s
15	*pLacUV5-thiC-87-175*	A138	-	14 ± 3 s
16	*pLacUV5-thiC-87-175*	C158	-	26 ± 7 s
17	*pLacUV5-thiC-87-175-NPOM*	C158	-	16 ± 3 s
18	*pLacUV5-thiC-87-175-NPOM-StemMut*	C158	-	18 ± 3 s
19	*pLacUV5-thiC-87-175-G159A*[1]	C158	-	16 ± 4 s
20	*pLacUV5-thiC-87-175-G159A-StemMut*[1]	C158	-	15 ± 5 s

[1]Values were calculated using a two-step kinetic model.

Supplementary Table 2. Initiator di- or trinucleotide, subset of unlabeled nucleotides and radio-labeled nucleotide used in transcription initiation of different templates.

Templates	Initiator di- or trinucleotide	Subset of unlabeled nucleotides	Radio-labeled nucleotide	EC length
pLacUV5-tbpA-92<br>*pLacUV5-tbpA(+5)-92*<br>*pLacUV5-tbpA(+5)-92(85-NPOM)*	GUU	ATP + CTP	$[\alpha\text{-}^{32}P]$ UTP	9 nt<br>14 nt<br>14 nt
pT7A1-tbpA-21-92(85-NPOM)<br>*pT7A1-tbpA-21-92(85-biotin)*	AUC	ATP + GTP	$[\alpha\text{-}^{32}P]$ CTP	20 nt<br>20 nt
pLacUV5-his-132-WT<br>*pLacUV5-his-132-G103A*<br>*pLacUV5-his-132-NPOM*<br>*pLacUV5-his-132-NPOM-StemMut*	AU	ATP + GTP	$[\alpha\text{-}^{32}P]$ CTP	20 nt
pLacUV5-his-62-132<br>*pLacUV5-his-62-132 NPOM*	None	ATP + UTP	$[\alpha\text{-}^{32}P]$ CTP	15 nt
pLacUV5-thiC-87-175-G159A<br>*pLacUV5-thiC-87-175-G159A-StemMut*<br>*pLacUV5-thiC-87-175*<br>*pLacUV5-thiC-87-175-NPOM*<br>*pLacUV5-thiC-87-175-NPOM-StemMut*	CA	ATP + GTP	$[\alpha\text{-}^{32}P]$ UTP	11 nt

Supplementary Table 3. DNA constructs used in this study.

Constructs	Oligonucleotides
***E. coli RNAP in vitro* transcription assays**	
pLacUV5-tbpA-92	275 AL<br>1579 JFN (Reverse primer)
pLacUV5-tbpA(+5)-92	2223 JFN (Forward primer)<br>1579 JFN (Reverse primer)
pLacUV5-tbpA(+5)-92(85-NPOM)	2392 JFN (Forward template)<br>NPOM SEQUENCE 4 (Reverse primer)
pT7A1-tbpA-21-92(85-NPOM)	3334 JFN (Forward template)<br>NPOM SEQUENCE 4 (Reverse primer)
pT7A1-tbpA-21-92(85-biotin)	3334 JFN (Forward template)<br>3344 JFN (Reverse primer)
pLacUV5-his-132-WT	3279 JFN (Forward template)<br>3280 JFN (Reverse primer)
pLacUV5-his-132-G103A	3281 JFN (Forward template)<br>3282 JFN (Reverse primer)
pLacUV5-his-132-NPOM	3281 JFN (Forward template)<br>3283 JFN (Reverse primer)
pLacUV5-his-132-NPOM-StemMut	3370 JFN (Forward template)<br>3372 JFN (Reverse primer)
pLacUV5-his-62-132	3406 JFN (Forward template)<br>3280 JFN (Reverse primer)
pLacUV5-his-62-132 NPOM	3492 JFN (Forward template)<br>3283 JFN (Reverse primer)
pLacUV5-thiC-87-175-G159A	3621 JFN (Forward template)<br>3575 JFN (Reverse primer)
pLacUV5-thiC-87-175-G159A-StemMut	3587 JFN (Forward template)<br>3575 JFN (Reverse primer)
pLacUV5-thiC-87-175	3006 AC (Forward primer)<br>3540 JFN (Reverse primer)

pLacUV5-thiC-87-175-NPOM	3621 JFN (Forward template)<br>3609 JFN (Reverse primer)
pLacUV5-thiC-87-175-NPOM-StemMut	3587 JFN (Forward template)<br>3609 JFN (Reverse primer)
**T7 RNAP *in vitro* transcription assays**	
pLacUV5-tbpA(+5)-97	2660 JFN (Forward template)<br>1579 JFN (Reverse primer)
pLacUV5-tbpA(+5)-97(90-NPOM)	2660 JFN (Forward template)<br>NPOM SEQUENCE 4 (Reverse primer)

Supplementary Table 4. Summary of oligonucleotides used in this study.

**Oligonucleotides**	**Sequences 5'-3'**
275 AL	GGGCACCCCAGGCTTTACACTTTATGCTTCCGGCTCGTATAATGTGT<br>GGGTTCTCAACGGGGTGCCAC
1579 JFN	5’-biotin-AGGAGCCTCAAATCCCTTCG
2223 JFN	GGGCACCCCAGGCTTTACACTTTATGCTTCCGGCTCGTATAATGTGT<br>GGGTTCAAACCTCAACGGGGTGCCAC
2392 JFN	GGGCACCCCAGGCTTTACACTTTATGCTTCCGGCTCGTATAATGTGT<br>GG**G**TT*CAAAC*CTCAACGGGGTGCCACGCGTACGCGTGCGCTGAGAA<br>AATACCCGTCGAACCTGATCCGGATAACGCCGGCGAAGGGATTTGA<br>GGCTCCT
2660 JFN	TGTAATACGACTCACTATA**G**CGGTT*C*AAA*C*CTCAACGGGGTGCCAC<br>GCGTACGCGTGCGCTGAGAAAATACCCGTCGAACCTGATCCGGATA<br>ACGCCGGCGAAGGGATTTGAGGCTCCT
3006 AC	GGGCACCCCAGGCTTTACACTTTATGCTTCCGGCTCGTATAATGTGT<br>GGCAAGAGTTAATCTGCTATCGCATCGC
3257 JG	GGGCACCCCAGGCTTTACAC
3279 JFN	GGGCACCCCAGGCTTTACACTTTATGCTTCCGGCTCGTATAATGTGT<br>GG**A**TCGAGAGGGACACGGGGGATCCTCTAGTTTATGACACGCGTTC<br>AATTTAAACACCACCATCATCACCATCATCCTGACTAGTCTTTCAGG<br>CGATGTGTGCTGGAAGACATTCAGATCTTCCAGTGGTGCAT
3280 JFN	ATGCACCACTGGAAGATCTGAATGTCTTCCAGCACACATCGCCTGA<br>AAGACTAGTC
3281 JFN	GGGCACCCCAGGCTTTACACTTTATGCTTCCGGCTCGTATAATGTGT<br>GG**A**TCGAGAGGGACACGGGGGATCCTCTAGTTTATGACACGCGTTC<br>AATTTAAACACCACCATCATCACCATCATCCTGACTAGTCTTTCAGG<br>CGATGTGTGCTAGAAGACATTCAGATCTTCCAGTGGTGCAT
3282 JFN	ATGCACCACTGGAAGATCTGAATGTCTTCTAGCACACATCGCCTGA<br>AAGACTAGTC
3283 JFN	ATGCACCACTGGAAGATCTGAATGTCTTC/iNPOM-<br>dT/AGCACACATCGCCTGAAAGACTAGTC

3334 JFN	TCCAGATCCCGAAAATTTATCAAAAAGAGTATTGACTTAAAGTCTAACCTATAGGATACTTACAGCC**A**TCGAGAGGGACACGGCGAATCACGCGTACGCGTGCGCTGAGAAAATACCCGTCGAACCTGATCCGGATAACGCCGGCGAAGGGATTTGAGGCTCCT
3344 JFN	AGGAGCC/iBio-dT/CAAATCCCTTCGCCGGCGTTAT
3370 JFN	GGGCACCCCAGGCTTTACACTTTATGCTTCCGGCTCGTATAATGTGTGG**A**TCGAGAGGGACACGGGGGATCCTCTAGTTTATGACACGCGTTCAATTTAAACACCACCATCATCACCATCATATTAACTAGTCTTTCAGGCGATGTGTGCTAGAAGACATTCAGATCTTCCAGTGGTGCAT
3372 JFN	ATGCACCACTGGAAGATCTGAATGTCTTC/iNPOM-dT/AGCACACATCGCCTGAAAGACTAGTTAATATG
3406 JFN	GGGCACCCCAGGCTTTACACTTTATGCTTCCGGCTCGTATAATGTGTGG**C**ATCACCATCATCCTGACTAGTCTTTCAGGCGATGTGTGCTGGAAGACATTCAGATCTTCCAGTGGTGCAT
3492 JFN	GGGCACCCCAGGCTTTACACTTTATGCTTCCGGCTCGTATAATGTGTGG**C**ATCACCATCATCCTGACTAGTCTTTCAGGCGATGTGTGCTAGAAGACATTCAGATCTTCCAGTGGTGCAT
3540 JFN	TCCAAAAAGTTAAGGACGTGGCTTG
3575 JFN	TCCAAAAAGTTAAGGATGTGGCTTG
3587 JFN	GGGCACCCCAGGCTTTACACTTTATGCTTCCGGCTCGTATAATGTGTGG**C**AAGAGTTAATCTGCTATCGCATCGCCCCTGCCCGCATCGTCTCTTGCTTCATCCGTCGTCTGACAAGCCACATCCTTAACTTTTTGGA
3609 JFN	TCCAAAAAGTTAAGGA/iNPOM-dT/GTGGCTTGTCAGACGACGGATG
3621 JFN	GGGCACCCCAGGCTTTACACTTTATGCTTCCGGCTCGTATAATGTGTGG**C**AAGAGTTAATCTGCTATCGCATCGCCCCTGCGGCGATCGTCTCTTGCTTCATCCGTCGTCTGACAAGCCACATCCTTAACTTTTTGGA
NPOM SEQUENCE 4	AGGAGCC/iNPOM-dT/CAAATCCCTTCGCCGGCGTTAT

Supplementary References

1. Larson, M. H. *et al.* A pause sequence enriched at translation start sites drives transcription dynamics in vivo. *Science (80-.).* **344**, 1042–1047 (2014).
2. Weixlbaumer, A., Leon, K., Landick, R. & Darst, S. A. Structural basis of transcriptional pausing in bacteria. *Cell* **152**, 431–441 (2013).
3. Kyzer, S., Ha, K. S., Landick, R. & Palangat, M. Direct *Versus* Limited-step Reconstitution Reveals Key Features of an RNA Hairpin-stabilized Paused Transcription Complex. *J. Biol. Chem.* **282**, 19020–19028 (2007).
4. Rio, D. C. Expression and purification of active recombinant T7 RNA polymerase from E. coli. *Cold Spring Harb. Protoc.* **2013**, (2013).
