# Supplementary material for: Site-specific photolabile roadblocks for the study of transcription elongation in biologically complex systems
Source: Commun Biol. 2022 May 12;5:457. doi: 10.1038/s42003-022-03382-0 (PMC9098449; doi:10.1038/s42003-022-03382-0)
Supplement: Supplementary file 3 — Description of Additional Supplementary Files [file 42003_2022_3382_MOESM3_ESM.pdf]

Description of Additional Supplementary Files

File name: Supplementary Data 1
Description: Source file for graphs Figs. 4 and 5.
